# Role of the Ubiquitin Ligase RNF149 in the Development of Rat Neonatal Gonocytes

**DOI:** 10.3389/fendo.2022.896507

**Published:** 2022-05-13

**Authors:** Gurpreet Manku, Chi-Chon Kong, Martine Culty

**Affiliations:** ^1^The Research Institute of the McGill University Health Centre, McGill University, Montreal, QC, Canada; ^2^The Departments of Medicine and Pharmacology & Therapeutics, McGill University, Montreal, QC, Canada; ^3^Department of Pharmacology and Pharmaceutical Sciences, School of Pharmacy, University of Southern California, Los Angeles, CA, United States

**Keywords:** germ cells, gonocytes, ubiquitin proteasome system, ubiquitin ligase, proliferation

## Abstract

Male reproductive function depends on the formation of spermatogonial stem cells from their neonatal precursors, the gonocytes. Previously, we identified several UPS enzymes dynamically altered during gonocyte differentiation. The present work focuses on understanding the role of the RING finger protein 149 (RNF149), an E3 ligase that we found to be strongly expressed in gonocytes and downregulated in spermatogonia. The quantification of RNF149 mRNA from postnatal day (PND) 2 to 35 (puberty) in rat testis, brain, liver, kidney, and heart indicated that its highest levels are found in the testis. RNF149 knock-down in PND3 rat gonocytes was performed to better understand its role in gonocyte development. While a proliferative cocktail of PDGF-BB and 17β-estradiol (P+E) increased both the expression levels of the cell proliferation marker PCNA and RNF149 in mock cells, the effects of P+E on both genes were reduced in cells treated with RNF149 siRNA, suggesting that RNF149 expression is regulated during gonocyte proliferation and that there might be a functional link between RNF149 and PCNA. To examine RNF149 subcellular localization, EGFP-tagged RNF149 vectors were constructed, after determining the rat testis RNF149 mRNA sequence. Surprisingly, two variant transcripts were expressed in rat tissues, predicting truncated proteins, one containing the PA and the other the RING functional domains. Transfection in mouse F9 embryonal carcinoma cells and C18-4 spermatogonial cell lines showed differential subcellular profiles of the two truncated proteins. Overall, the results of this study support a role for RNF149 in gonocyte proliferation and suggest its transcription to variant mRNAs resulting in two proteins with different functional domains. Future studies will examine the respective roles of these variant proteins in the cell lines and isolated gonocytes.

## Introduction

Spermatogenesis is a process that encompasses numerous steps including phases of quiescence, proliferation, differentiation, migration, and apoptosis to ensure the production of sperm throughout the lifetime of a male (1). At the origin of sperm formation is the existence of a pool of germline stem cells, the spermatogonial stem cells (SSCs), originating from neonatal precursors, the gonocytes, also known as pre- or pro-spermatogonia (1, 2). We have previously shown that rat neonatal gonocytes undergo proliferation in response to platelet-derived growth factor (PDGF)-BB (P) and 17β-estradiol (E) while activating both the PDGF receptor (PDGFR) and Estrogen Receptor (ER) signaling pathways (3, 4). Furthermore, we have shown that gonocyte differentiation is induced by retinoic acid (RA) with activation of the PDGFR, JAK2, STAT5, and SRC signaling pathways (5, 6). While studying genes involved in gonocyte apoptosis, we also found that pro-apoptotic genes Cycs and Gadd45α were significantly upregulated in differentiating gonocytes, indicating their possible role in gonocyte apoptosis, a necessary step for eliminating gonocytes that failed to differentiate to spermatogonia (7). Studies have suggested that improper development of gonocytes can lead to the formation of testicular germ cell tumors (TGCTs), the most common type of cancer in young men (8, 9). Testicular cancer rates have been steadily increasing for the past few decades for reasons that are not completely known (10) and as a result, a better understanding of gonocyte development can help provide a more thorough insight into how testicular tumors form and why the incidence has been increasing.

We have previously shown that the ubiquitin proteasome system (UPS) is involved in gonocyte differentiation and that proteasome inhibition significantly reduced RA-induced gonocyte differentiation (11). UPS is the main pathway by which proteins are degraded in eukaryotes, involving a succession of enzymatic reactions involved in the attachment of an ubiquitin chain to a substrate protein targeted for degradation, including an E1 activating enzyme, an E2 conjugating enzyme, and an E3 ligase (12, 13). UPS activity regulates a variety of developmental and biological functions, such as myogenesis, bone formation and immune function (14–16). During the ubiquitination process, the E1 activating enzyme activates the ubiquitin molecule which is then transferred to the E2 conjugating enzyme and finally to an E3 ligase. The E3 ligase attaches the ubiquitin tag to the substrate to be targeted, which is then recognized by the 26S proteasome and degraded. Deubiquitinating enzymes modulate the pathway by removing ubiquitin molecules as needed (17, 18). Besides its major role in protein degradation, the UPS has been shown to be involved in other processes including signal transduction, kinase activation, cell cycle progression, cell proliferation, and protein interaction regulation (12, 13, 19, 20), while also playing an important role in the later stages of spermatogenesis (21). Given that spermatogenesis occurs *via* multiple tightly timed biological events (1), it is not surprising that the UPS is involved in this process, as spermatogenesis requires a large amount of protein turnover and degradation. Our study of UPS gene and protein expression in rat PND3 gonocytes and PND8 spermatogonia showed that the E3 ubiquitin ligase RNF149 (also known as DNA polymerase-transactivated protein 2; DNAPTP2) was downregulated in spermatogonia compared to neonatal gonocytes, as well as in gonocytes that had undergone RA-induced differentiation compared to undifferentiated germ cells of the same age (11). This suggested a likely role of RNF149 in neonatal gonocyte development. The present study further examined the role of E3 ubiquitin ligase RNF149 in gonocyte development, revealing its involvement in cell proliferation, and the existence of two variant forms of the protein with distinct functional moieties observed in different subcellular compartments.

## Materials and Methods

### Animals and Tissue Collection

Newborn male Sprague Dawley rats obtained from Charles Rivers Laboratories (Saint-Constant, QC, CA). Rats aged from PND2 to PND35 were euthanized and handled according to protocols approved by the McGill University Health Centre Animal Care Committee and the Canadian Council on Animal Care. Several organs, including brain, heart, liver, kidneys and testes, were collected and either frozen for gene expression analysis by quantitative Real Time PCR analysis (qPCR), or fixed in 4% paraformaldehyde for immunohistochemical analysis.

### Gonocyte Isolation

Gonocytes were isolated from PND3 rat testes following a well-established protocol as previously described (3, 4, 6). In short, testes from 40 rats were isolated and decapsulated. Gonocytes were isolated by sequential tissue enzymatic digestion and differential plating overnight in RPMI 1640 medium (Invitrogen, Thermo Fisher Scientific, ON, CA) with 5% fetal bovine serum (FBS) (Invitrogen), 2% penicillin/streptomycin (CellGro, Manassas, VA, USA), and 1% amphotericin B (CellGro). During overnight plating, somatic cells adhered to the culture plates while germ cells remained non-adherent. The next day, non-adherent germ cells were further separated using a 2-4% bovine serum albumin (BSA) (Roche Diagnostics, Indianapolis, IN, USA) gradient. Gonocytes were judged by morphology and larger size compared to Sertoli/myoid cells by phase contrast microscopy. Fractions containing the most gonocytes were pooled, centrifuged, and collected for treatments or RNA analysis with a purity of at least 85%. Cell viability was assessed by trypan blue exclusion assay, together with live gonocyte quantification on hemacytometer. The enrichment efficacy, viability, and identity of the gonocytes were validated using a variety of approaches in previous studies (3, 7, 11, 22). Samples with lower purity were used for immunocytochemical analysis. All experiments were performed using a minimum of three independent gonocyte preparations.

### RNF149 Silencing and Gonocyte Treatment

For RNF149 silencing, after gonocyte collection from BSA gradient, cells were plated at a density of 10000 cells/well in a 24-well plate using RPMI 1640 media free of antibiotics, 2.5% FBS, and amphotericin B, as they are not recommended while using siRNA protocols. Gonocytes were then treated with Mock, Scrambled (10nM), or RNF149 silencing duplexes (at various concentrations) (siRNA TriFECTa Kit, IDT Inc., San Jose, CA, USA) using Lipofectamine RNAiMAX (Invitrogen) and Opti-MEM transfection medium (Invitrogen). The three silencing duplexes used were: (1) Sense Strand: 5’-GGAAUUGUGAAAUGUAGUUCCUUAT-3’, Antisense Strand: 5’-AUAAGGAACUACAUUUCACAAUUCCAC-3’, (2) Sense Strand: 5’-ACCUGUAAAGUGAGAAAUCUUGCCA-3’, Antisense Strand: 5’-UGGCAAGAUUUCUCACUUUACAGGUUC-3’, (3) Sense Strand: 5’-GGAAACUAAGAAGGUUAUUGGCCAG-3’, Antisense Strand: 5’- CUGGCCAAUAACCUUCUUAGUUUCCUU-3’. A red fluorescent dye was transfected at 10nM and served as a positive control. Cells were transfected for 48 hours and were then treated with or without PDGF-BB (Sigma Aldrich, Oakville, ON, CA) and 17β-estradiol (Sigma Aldrich) for an additional 24 hours. This additional 24 hour treatment contained 2.5% FBS and antibiotics. Cells were then collected for RNA analysis and immunocytochemical analysis on microscopic slides.

### F9 Mouse Embryonal Teratocarcinoma Cell Culture

As previously described, F9 cells were maintained in DMEM medium (Invitrogen) containing 10% fetal bovine serum (FBS) (Invitrogen) at 37°C and 5.0% CO_2_ (5, 6). Cells were plated on gelatin-coated culture dishes on day 1 and treated on day 2. Cells were plated at a density of 30000 cells/well in a 6-well plate using DMEM media free of antibiotics, FBS, and amphotericin B, as they are not recommended while using siRNA protocols. Cells were then treated with Mock, Scrambled (10nM), or RNF149 silencing duplexes (20nM/duplex) (siRNA TriFECTa Kit, IDT Inc., San Jose, CA, USA) using Lipofectamine RNAiMAX (Invitrogen) and Opti-MEM transfection medium (Invitrogen). The three silencing duplexes used were: (1) Sense Strand: 5’- GGCAUACAGUAAUGUCUUUAAAUGA-3’, Antisense Strand: 5’- UCAUUUAAAGACAUUACUGUAUGCCUA-3’, (2) Sense Strand: 5’- AGCGGAGACUGUAGAACUUGGAAAT-3’, Antisense Strand: 5’- AUUUCCAAGUUCUACAGUCUCCGCUCA-3’, (3) Sense Strand: 5’- CGCGGGAACAGGAAACAUAGUCGTC-3’, Antisense Strand: 5’- GACGACUAUGUUUCCUGUUCCCGCGUG-3’. A red fluorescent dye was transfected at 10nM and served as a positive control. Cells were transfected for 48 hours and were then treated with or without Glial Cell-Derived Neurotrophic Factor (GDNF, 100ng/ml, Millipore, Etobicoke, ON, CA), Fibroblast Growth Factor (FGF2, 10ng/ml, Millipore) and GDNF Family Receptor 1A (GFRα1, 300ng/ml, R&D Systems, Minneapolis, MN, USA) to promote proliferation or retinoic acid (RA, 10^-7^M, Sigma Aldrich) to promote differentiation for an additional 24 hours. This additional 24 hour treatment contained 10% FBS and antibiotics. Cells were then collected for RNA analysis.

### C18-4 Mouse Spermatogonia Cell Culture

C18-4 cells (a gift from MC Hofmann, Houston TX, USA) were maintained in DMEM medium (Invitrogen) containing 10% fetal bovine serum (FBS) (Invitrogen) at 34°C and 5.0% CO_2_. Cells were plated on day 1 and treated on day 2 similarly to how F9 cells were treated above.

### RNA Extraction and cDNA Synthesis

Total RNA was extracted from cell pellets using the PicoPure RNA isolation kit (Arcturus, Mountain View, CA, USA) and digested with DNase I (Qiagen, Valencia, CA, USA) as previously described (11). Tissue total RNA of testis and other organs in PND2 to PND35 rat pups were extracted using QIAGEN RNAeasy Mini kit (Qiagen, Santa Clarita, CA) as previously described (19). For quantitative PCR (qPCR) analysis, cDNA was synthesized from the extracted RNA by using the single strand cDNA transcriptor synthesis kit (Roche Diagnostics) following the manufacturer’s instructions.

### Reverse Transcriptase (RT)-PCR Analysis

RNF149 gene expression in various tissue samples was examined by PCR and gel electrophoresis. Two primer sets were used: (1) RNF149 cloning primers (Reverse, 5’- CGAGCGGTCTCACTCTTCC-3’; Forward: 3’-TGAGGCTGTCAATGAAGACG-5’), and variant (VA) form testing primers (Reverse: 5’-AAGGAATTCCAGTAAAAATGAGG; Forward: 3’-TTAAAGTTTTCAATACACACTGC-5’). PCR reactions were carried out using GoTaq^®^ DNA polymerase (Promega, Madison, WI, USA) and amplified using the iCycler thermal cycler (Bio-Rad, Hercules, CA, USA). PCR cycle conditions: 95°C for 3 min; 45 cycles of 95°C for 60 sec, 55°C for 60 sec, and 72°C for 2 min; followed by a 10 minute extension at 72°C and a cool down to 4°C. PCR products were then run alongside molecular weight standards (New England BioLabs, Whitby, ON, CA) on a 1.5% agarose gel. Gel densitometry analysis was performed using Multi-Gauge software (FujiFilm, Mississauga, ON, CA).

### Quantitative Real Time PCR (qPCR)

QPCR was performed using a LightCycler 480 with a SYBR Green PCR Master Mix kit (Roche Diagnostics) as previously described (6, 22). The primer sets used were designed using the Roche primer design software (Roche Diagnostics) and are listed in **Table 1**. QPCR cycling conditions: initial step at 95°C followed by 45 cycles at 95°C for 10 sec, 61°C for 10 sec, and 72°C for 10 sec. The comparative threshold cycle (C_t_) method was used to analyze the data and 18S rRNA was used for data normalization. We initially determined the Ct values of three potential housekeeping genes, GAPDH, Tubulin, and 18S rRNA in cDNA samples from isolated gonocytes cultured for 1 day after siRNA interference, and 18S rRNA showed that it presented minimal changes in Ct values between samples. Assays were performed in triplicate. All experiments were performed using a minimum of three independent sample preparations and the mean ± SEM are plotted.

**Table 1:** Quantitative real time PCR Primers.

Species	Gene	Forward Primer	Reverse Primer
Rat	Rnf149	TGCACCTTCAAGGACAAGGT	GCGCTCCTGGTTGTAGACC
Rat	Pcna	CGTAGTATCACCAGATGGCATCTTTA	GGACTTAGACGTTGAGCAACTTGG
Rat	Ccnb2	AAAACCTCACCAAGTTCATCG	GAGGGATCGTGCTGATCTTC
Rat	18S	cgggTGCTCTTAGCTGAGTGTCCcG	CTCGGGCCTGCTTTGAACAC
Mouse	Rnf149	CGGTCAGTCTGTGGTGTTTG	CCTTCTTAGTCTCCTTCCTATGATTC
Mouse	Stra8	CTCTCCCACTCCTCCTCCACTC	CGGTATTGCTTGTAAAAGTTGAGATA
Mouse	18S	CGGAATCTTAATCATGGCCTCAGTTC	ACCGCAGCTAGGAATAATGGAAT

### Immunohistochemistry

RNF149 protein expression was determined in paraformaldehyde fixed, paraffin-embedded sections of PND2-35 testes and PND3 and PND10 brain, heart, liver, and kidney sections. Slides were stained using previously described methods (22). In brief, slides were first dewaxed and rehydrated using Citrosolv (Fisher Scientific, Toronto, ON, CA) and Trilogy solution (Cell Marque IVD, Rocklin, CA, USA). Following treatment with Dako Target Retrieval solution (DAKO, Burlington, ON, CA), the sections were incubated with PBS (Invitrogen) containing 10% goat serum (Vector Laboratories, Burlington, ON, CA), 1% BSA (Roche Diagnostics) and 0.02% Triton X100 (Promega) for one hour to block non-specific protein interactions. Slides were subjected to a 30% hydrogen peroxide/methanol solution incubation. The sections were then treated with the RNF149 antibody (Santa Cruz, Dallas, TX, USA) diluted in PBS (Invitrogen) containing 1% BSA (Roche Diagnostics) and 0.02% Triton X100 (Promega) overnight at 4°C. Once the overnight incubation was complete, sections were incubated with biotin-conjugated goat anti-rabbit secondary antibody (BD Pharmingen, San Jose, CA, USA) diluted in PBS (Invitrogen) containing 1% BSA (Roche Diagnostics) for 60 minutes at room temperature. Immunoreactivity was detected using streptavidin-peroxidase (Invitrogen) and AEC single use solution (Invitrogen). The sections were then counter-stained with hematoxylin (Sigma Aldrich), coated with Crystal Mount (Electron Microscopy Sciences, Hatfield, PA, USA) and dried, and then cover-slipped using Permount (Fisher, Thermo Scientific) and glass coverslips (Fisher Scientific). Slides were then examined under bright-field microscopy with a BX40 Olympus microscope (Olympus, Center Valley, PA, USA) coupled to a DP70 Olympus digital camera (Olympus). Negative controls were performed by incubating sections with pre-immune Rabbit IgG (Invitrogen).

### Immunocytochemistry

Microscopic slides were prepared on a cytospin centrifuge using Mock, Scrambled, and siRNA treated gonocytes (with or without PDGF-BB and 17β-estradiol) for protein analysis. C18-4 cells (detailed above) were also cultured in 8-well chamber slides (BD Falcon, Oakville, ON, CA) and analyzed using immunocytochemistry. The protocol used for immunocytochemistry, as previously described, was as follows (11). In brief, slides were treated with Dako Target Retrieval solution (DAKO) and then blocked with PBS (Invitrogen) containing 10% goat serum (Vector Laboratories), 1% BSA (Roche Diagnostics) and 0.02% Triton X100 (Promega) for one hour to block non-specific protein interactions. Slides were then incubated with the phospho-ERK antibody (Cell Signaling, Danvers, MA, USA) for gonocyte analysis and the RNF149 and PCNA antibodies (Santa Cruz) for C18-4 cell analysis. Antibodies were diluted in PBS (Invitrogen) containing 1% BSA (Roche Diagnostics) and 0.02% Triton X100 (Promega) overnight at 4°C. Once the overnight incubation was complete, the slides were incubated with a biotin-conjugated goat anti-rabbit or anti-mouse secondary antibody (BD Pharmingen) diluted in PBS (Invitrogen) containing 1% BSA (Roche Diagnostics) for one hour at room temperature. Immunoreactivity was then detected using a combination of streptavidin-peroxidase (Invitrogen) and AEC single use solution (Invitrogen). Slides were counter-stained with hematoxylin (Sigma Aldrich), coated with Crystal Mount (Electron Microscopy Sciences) and dried, and then cover-slipped using Permount (Thermo Scientific) and glass coverslips (Fisher Scientific). The slides were then viewed using a BX40 Olympus microscope (Olympus, Center Valley, PA, USA) coupled to a DP70 Olympus digital camera (Olympus). For RNF149 silencing analysis, phospho-ERK positive gonocytes were easily distinguished from the remaining somatic cells by their much larger size on the cytospin slides. They were counted and their number was normalized to the total gonocyte number for each treatment condition, and the data means plotted as percent of the total gonocyte numbers.

### Recombinant DNA Constructs and Amplification

Template RNF149 cDNA was cloned from PND3 testis total cDNA with RNF149 cloning primers by GoTaq^®^ DNA polymerase (Promega) to create poly-A tailing. PCR products were then separated and extracted from 1.5% agarose gels. Purified segments were then ligated to pGEM^®^-T Easy Vector System I (Promega) and transformed into DH5-α competent cells (Invitrogen) overnight at 37°C. Single colonies were collected and cultured in LB (Invitrogen) for 8 hours. Plasmids were purified by QIAprep Spin Miniprep kit (Qiagen) and sent for sequencing (Genome Quebec, Montreal QC, CA). After sequencing, two confirmed variant forms of RNF149 were then further amplified and purified with HiSpeed Plasmid Maxi Kit (Qiagen). These two variant forms were ligated into pEGFP-N1 and pEGFP-C2 (Clontech, Mountain View, CA, USA). Based on gene maps constructed using SnapGene^®^ software (Version 2.8, GSL Biotech, Chicago, IL, USA), restriction sites were selected at HindIII and KpnI with the 2.1 buffer (New England BioLabs). The following constructs were used: N-terminal EGFP-tagged VA1 (pPA-EGFP) and VA2 (pRING-EGFP), C-terminal EGFP-tagged VA1 (pEGFP-PA) and VA2 (pEGFP-RING). Gene maps of RNF149-EGFP plasmids are shown as **Supplementary Figures 1**, **2**.

### Transfections and Live Cell Imaging

Both cell lines were grown on 35mm fluoro-dish cell culture dishes (World Precision Instruments, Sarasota, FL, USA) at a cell density of 25 million cells/dish before transfection. Cells were transfected with the plasmids mentioned above with either Set 1: BFP-KDEL (Blue ER tubule marker, Addgene, Cambridge, MA, USA) and DsRed-Mito (Red mitochondria marker, Clontech) or Set 2: pDsRed2-ER (Red ER marker, Clontech), one day before confocal microscopy observation, using Lipofectamine™ 3000 (Invitrogen) according to the manufacturer’s protocol. LysoTracker Blue DND-22 (60nM, Life Technologies) is added to Set 2 cells before observation for 30 minutes. Before observation under confocal microscope, cells are gently washed with culture medium, and then 1ml Opti-MEM medium (Life Technologies) is added to replace culture medium. Cell samples were analysed by Zeiss LSM880 Laser Scanning Confocal and Super-Res SIM/PALM/dSTORM system (Zeiss) at the McGill University Health Centre Research Institute Molecular Imaging Core Facility. Images were collected over a 60 minute time period.

### Statistical Analysis

Statistical analysis was performed using an unpaired two-tail Student’s t-test using statistical analysis functions in the GraphPad Prism 5.0 program (GraphPad Inc., San Diego, CA, USA). All experiments were performed where N equals a minimum of three independent experiments. A P-value less than 0.05 was considered statistically significant.

## Results

### RNF149 Expression Profile in Neonatal to Pubertal Rat Organs

As previously mentioned, we have shown that RA-induced gonocyte differentiation requires an active ubiquitin proteasome system (UPS), and identified a number of UPS genes and proteins differentially expressed between PND3 gonocytes and PND8 spermatogonia (11). Amongst those identified, RNF149 was found to be more abundant in gonocytes than spermatogonia, suggesting that this UPS gene is decreased during the process of differentiation and remains low thereafter. In order to confirm this hypothesis and to understand the role of RNF149 in rat development, tissues such as testis, kidney, liver, heart, and brain were collected from rat pups aged from PND2 to PND35 for gene expression and immunohistochemistry studies.

**Figure 1:**
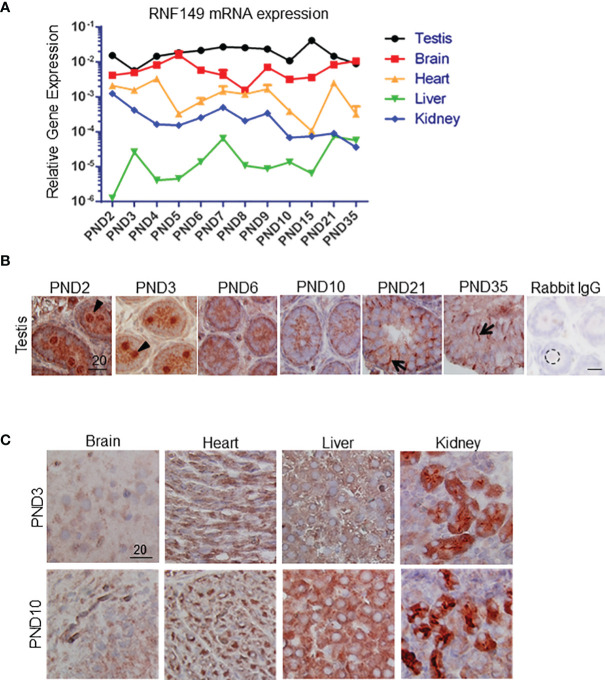
RNF149 expression profile in various organs in rats at various ages. **(A)** RNF149 mRNA levels in various tissues from PND2 to PND35. **(B)** RNF149 protein expression in testes from PND2 to PND35. Arrowhead: germ cell; arrow: Sertoli cell. **(C)** RNF149 expression in brain, heart, liver, and kidney at PND3 and PND10. 20μm scale shown. Representative images shown.

Using qPCR analysis, we found that RNF149 mRNA expression was highest in the testis at all ages analyzed when compared to RNF149 mRNA expression levels in the brain, heart, liver, and kidney at the same ages (**Figure 1A**). In neonatal testes, RNF149 was mainly expressed in gonocytes and was found in Sertoli cells only at older ages (**Figure 1B**). Interestingly, RNF149 was found highly expressed in the nucleus of PND2 and PND3 gonocytes, with a weaker staining in gonocyte cytoplasm. RNF149 also appeared to translocate to the cytoplasm in spermatogonia (**Figure 1B**). Although gene expression levels of RNF149 in other organs were lower than in the testis, we also observed weak RNF149 staining in the brain, liver, and heart at PND3, a more robust staining in the PND10 liver and a strong signal in cells of kidney tubules at both ages (**Figure 1C**). This suggests that RNF149 might play an important role in regulating the development of different tissues, mainly testis and kidney.

### RNF149 Silencing Leads to Reduced Cell Proliferation in Neonatal Gonocytes

**Figure 2:**
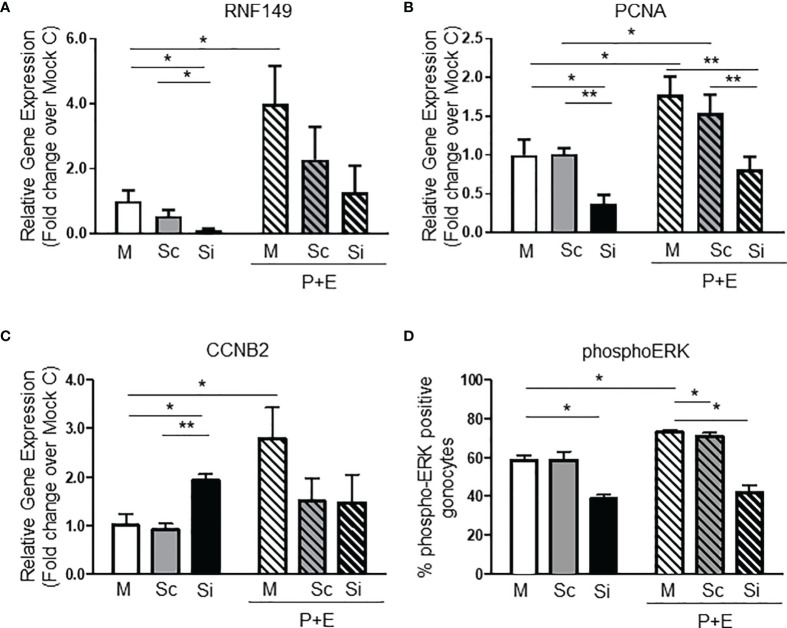
RNF149 knockdown in PND3 gonocytes. **(A)** RNF149 mRNA expression in gonocytes first treated with mock (M), scrambled siRNA (Sc), or RNF149 siRNA (Si) for 48 hours and then treated with or without PDGF-BB (10^-9^M) and 17β-estradiol (10^-6^M) [with 2.5% fetal bovine serum (FBS)] for an additional 24 hours. Results shown are from N=3-5 independent germ cell preparations (each done in duplicate) and are plotted mean ± SEM. *p-value<0.05. **(B)** PCNA mRNA expression and **(C)** CCNB2 mRNA expression in similarly treated cells. **p-value<0.01. **(D)** Treated gonocytes were immunostained using phospho-ERK antibody. Total number of gonocytes and positively stained cells were counted. Percentage of phospho-ERK-positive cells was graphed. Results shown are from N=3 independent germ cell preparations. *p-value<0.05.

To understand the possible mechanism of RNF149 in regulating gonocyte development, we started by testing the function of RNF149 in gonocyte proliferation. RNF149 mRNA was efficiently knocked down after 48 hours of treatment with a triad of siRNA duplexes using Lipofectamine transfection (**Figure 2A**). Compared to mock treatment conditions and those of scrambled, there was a significant decrease in *Rnf149* expression in gonocytes treated with siRNA, indicating an efficient knockdown (**Figure 2A**). We have previously shown that a combination of PDGF-BB (P) and 17β-estradiol (E) induces gonocyte proliferation. Here, we found that when gonocytes were treated with P+E, there was a significant increase in *Rnf149* mRNA expression in the mock treated cells, indicating a possible role of RNF149 in gonocyte proliferation. When analyzing Proliferating Cell Nuclear Antigen (*Pcna*) mRNA levels as a marker for gonocyte proliferation, we found that silencing RNF149 led to a significant decrease in proliferation (**Figure 2B**). Furthermore, as expected, the addition of P+E to gonocytes in the mock or scrambled conditions significantly upregulated *Pcna* expression. We also found that when P+E was added to the treated cells, siRNA-treated gonocytes had significantly lower *Pcna* expression compared to Mock cells treated with P+E, indicating that regardless of P+E stimulation, silencing RNF149 had a negative effect on proliferation. To confirm these findings, we also analyzed cyclin B2 (*Ccnb2*) mRNA expression (**Figure 2C**), which is another marker used to assess cell proliferation as it is an essential part of the cell cycle regulatory machinery involved in controlling the G2/M transition (23). However, unlike PCNA, we found that *Ccnb2* mRNA levels were significantly upregulated upon RNF149 silencing, although *Ccnb2* induction by P+E was reduced upon RNF149 silencing, suggesting that RNF149 disrupts *Ccnb2* expression in basal and proliferative conditions. Taken together, the analysis done on PCNA and CCNB2 gene expression indicates a complex and dynamic role of RNF149 in gonocyte proliferation.

To further explore the role of RNF149 in gonocyte proliferation we analyzed the activation of ERK, since we had previously shown that the MEK/ERK signalling pathway is involved in gonocyte proliferation (4). Thus, we examined whether there were any changes in levels of ERK phosphorylation upon P+E treatments in control cells and cells in which RNF149 was knocked down with siRNA. Gonocytes were immunostained to determine the levels of phosphorylated ERK, and the number of gonocytes positively stained for phospho-ERK in each condition was determined. As expected, there was a significant increase in phospho-ERK-positive gonocytes treated with P+E compared to Mock gonocytes alone (**Figure 2D**). Furthermore, there was a decrease in ERK activation when RNF149 was silenced in gonocytes with or without P+E treatment, similarly to *Pcna* mRNA expression. Taken together, these data suggest that when RNF149 is knocked down, there is a significant decrease in the expression of markers for gonocyte proliferation, further supporting its role in gonocyte proliferation.

### RNF149 Knockdown in C18-4 Mouse Spermatogonia Cells Does Not Affect Proliferation or Differentiation

**Figure 3:**
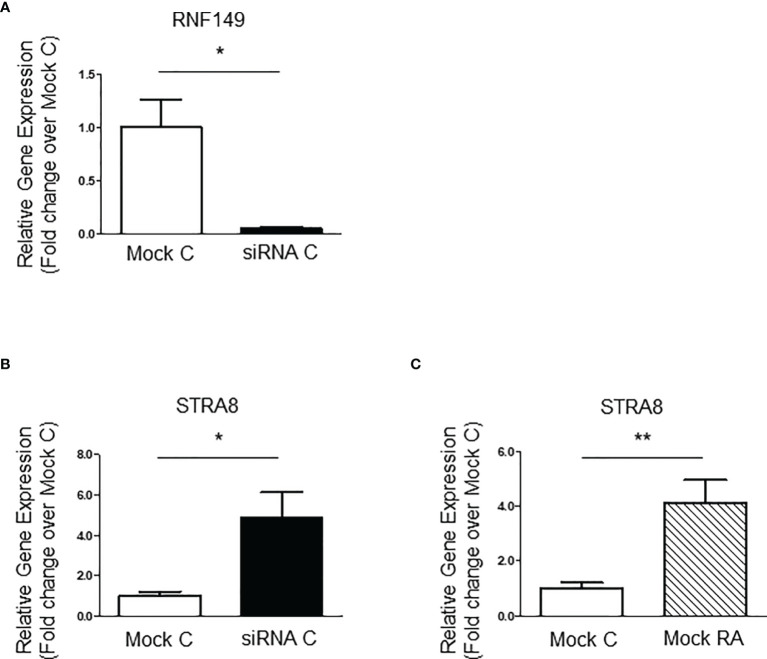
RNF149 knockdown in C18-4 cells. **(A)** RNF149 mRNA expression in C18-4 cells treated with mock and siRNA for 48 hours. Results shown are from N=3 independent cell passages and are plotted mean ± SEM. *p-value<0.05. **(B)** Stra8 mRNA expression in C18-4 cells treated with mock and siRNA for 48 hours. *p-value<0.05. **(C)** Stra8 mRNA expression in mock cells treated with or without RA (10^-7^M) for an additional 24 hours. **p-value<0.01.

Although there is no cell line model available for gonocytes, the mouse-derived C18-4 spermatogonia cell line is commonly used to study type A- spermatogonial development (24). Here, we used C18-4 cells to determine whether RNF149 knockdown had a similar effect on spermatogonial development. After confirming efficient RNF149 mRNA knockdown by qPCR (**Figure 3A**), we found that upon RNF149 mRNA silencing, there was a significant increase in Stra8 mRNA levels (**Figure 3B**). Stra8 mRNA level increases were also seen when Mock C18-4 cells were treated with RA (**Figure 3C**), confirming its use as a marker for spermatogonial differentiation. Unlike gonocytes, there was no significant change in the mRNA levels of PCNA upon RNF149 silencing (data not shown). Furthermore, when treating C18-4 cells with a cocktail of glial-cell derived neurotrophic factor (GDNF), fibroblast growth factor (FGF2), and GDNF family receptor alpha 1 (GFRα1), known to promote proliferation (25), there was no significant change seen in PCNA mRNA expression (data not shown). Taken together, this data indicates that unlike in gonocytes, RNF149 is likely negatively involved in spermatogonial differentiation, and not proliferation.

### RNF149 Silencing in F9 Mouse Embryonal Teratocarcinoma Cells Does Not Affect Their Proliferation or Differentiation

**Figure 4:**
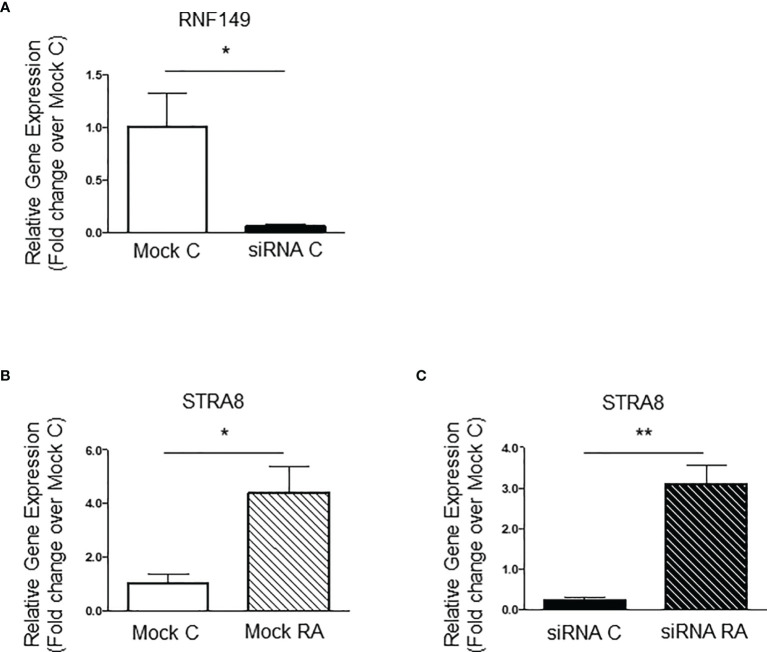
RNF149 knockdown in F9 cells. **(A)** RNF149 mRNA expression in F9 cells treated with mock and siRNA for 48 hours. Results shown are from N=3 independent cell passages and are plotted mean ± SEM. *p-value<0.05. **(B)** Stra8 mRNA expression in F9 cells in mock cells treated with or without RA (10^-7^M) for an additional 24 hours. *p-value<0.05. **(C)** Stra8 mRNA expression in siRNA treated cells also treated with or without RA (10^-7^M) for an additional 24 hours. **p-value<0.01.

Our lab previously showed that F9 cells, considered as surrogate for embryonic stem cells, also share similar traits with gonocytes, especially in their ability to express the spermatogonial marker STRA8 in responses to RA treatment, and in the existence of crosstalk between RA and PDGFR signaling pathways (6). Moreover, F9 cells proliferate in response to PDGF-AA, similarly to gonocytes that proliferate in response to PDGF-BB. Due to their similarities, F9 cells can be used as a model for the study of gonocyte differentiation. Here, we used F9 cells to determine whether RNF149 knockdown had a similar effect on these cells as it did on gonocyte proliferation. Upon confirmation of efficient RNF149 mRNA knockdown (**Figure 4A**), we found that independent of F9 cells being treated with siRNA or not, there was a significant increase in Stra8 mRNA levels upon RA treatment, as expected (**Figures 4B, C**). Silencing RNF149 mRNA had no significant effect on F9 cell proliferation also (data not shown). Furthermore, there was no significant change seen in PCNA mRNA expression in cells where RNF149 was silenced (data not shown). Taken together, these data suggest that although there are many similarities between the development of F9 cells and gonocytes, the involvement of RNF149 in their proliferation is not a common characteristic between these two cell types.

### Two Variant Forms of RNF149 Transcripts Are Expressed in Rat Tissues

**Figure 5:**
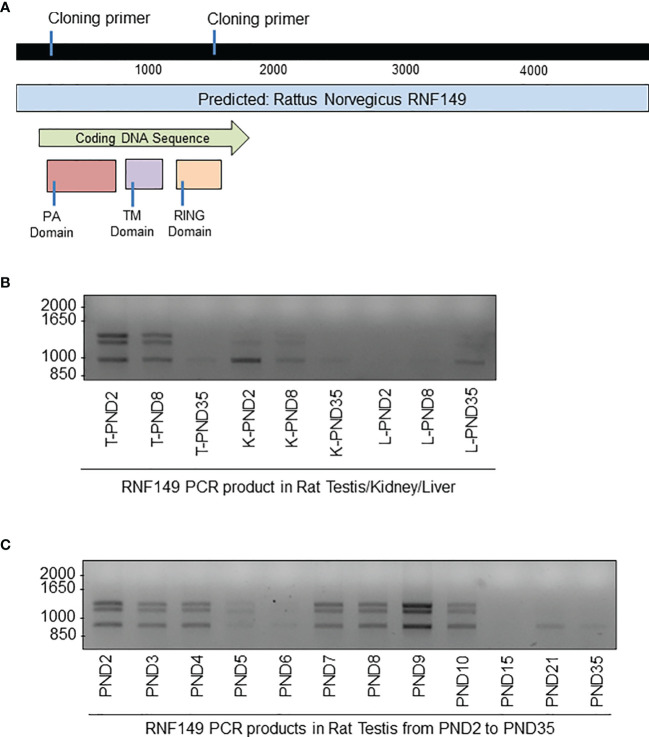
PCR analysis of RNF149 sequences cloned from tissues of rat pups at different ages. **(A)** Gene map of RNF149. Blue bar represents total predicted gene, green bar represents the RNF149 coding sequence that includes the PA domain (red), transmembrane TM domain (purple), and RING domain (orange). **(B)** PCR products of RNF149 in rat testes at various ages. **(C)** PCR products of RNF149 in various rat organs and various ages. T, testis; K, kidney; L,liver.

Given that there is limited information about RNF149 and its possible role in germ cell development, we proposed to build EGFP-tagged RNF149 vectors to better understand its cell localization and mechanism of action (**Figure 5A**). Interestingly, with primers designed based on a predicted sequence that would generate a single product approximately 1185bp in size, three major variant forms were found in all tissue sample examined, showing stronger expression in the testis and kidney than in the liver (**Figure 5B**). This finding was in agreement with both the qPCR and tissue staining results presented above. Furthermore, the three major variant forms were also present in the rat testes at ages PND2 to PND35 at varying intensities (**Figure 5C**).

Two PCR products that were approximately 1000bp in size were then cloned into pGEM^®^-T Easy Vectors for sequencing. The largest band obtained was 1400bp but because it was much larger than the expected size of full length RNF149 (1185bp), this band was not further used. However, one cannot exclude that it may correspond to a true RNF149 variant mRNA with intron retention, as found for a number of germ cell and cancer variant transcripts.

**Figure 6:**
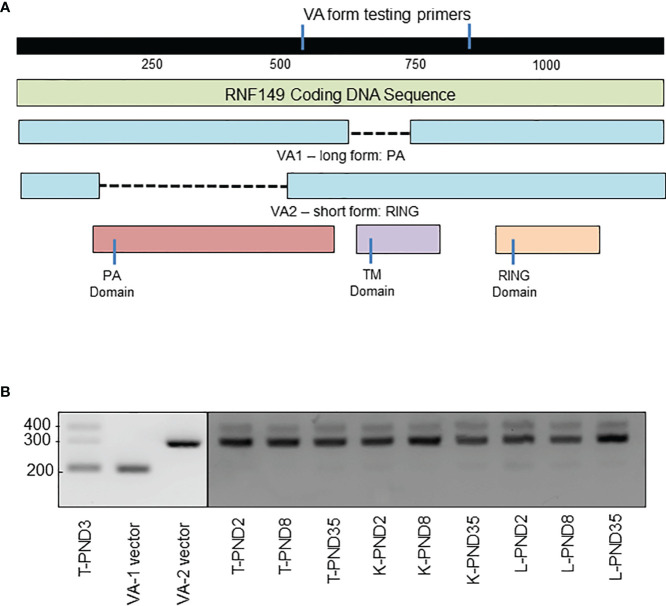
PCR analysis of RNF149 variant forms in rat testes, kidney, and liver at various ages. **(A)** Gene map of RNF149 coding sequence. Green bar represents total RNF149 coding sequence that includes the PA domain (red), transmembrane TM domain (purple), and RING domain (orange). Blue bars represent two variant forms of RNF149 found in the rat. **(B)** PCR products of RNF149 in PND3 rat testes, and VA1 and VA2 plasmids compared to kidney and liver at various ages. T, testis; K, kidney; L, liver.

Sequencing results indicated that there are two variant forms in rat tissues: VA1 (1066bp) that expresses the PA domain and VA2 (862bp) that expresses the TM-RING domain (**Figure 6A**). Although a great number of mutated RNF protein expressions have been found in cancer patients, these might be the first naturally expressed variant forms of RNF proteins in rat. Based on sequencing results, a primer set was designed to verify the expression of these two variant forms in other organs, which for VA1 would produce a 182bp product, and for VA2, a 301bp product. The results showed that almost all organs tested had three bands, two of which matched our predictions (**Figure 6B**). Taken together, this data suggests the presence of variant RNF149 forms present in the rat testis. The exact functions of these variants remain to be elucidated.

### Subcellular Localization of EGFP-Tagged RNF149 Isoforms in C18-4 and F9 Cell Lines

**Figure 7:**
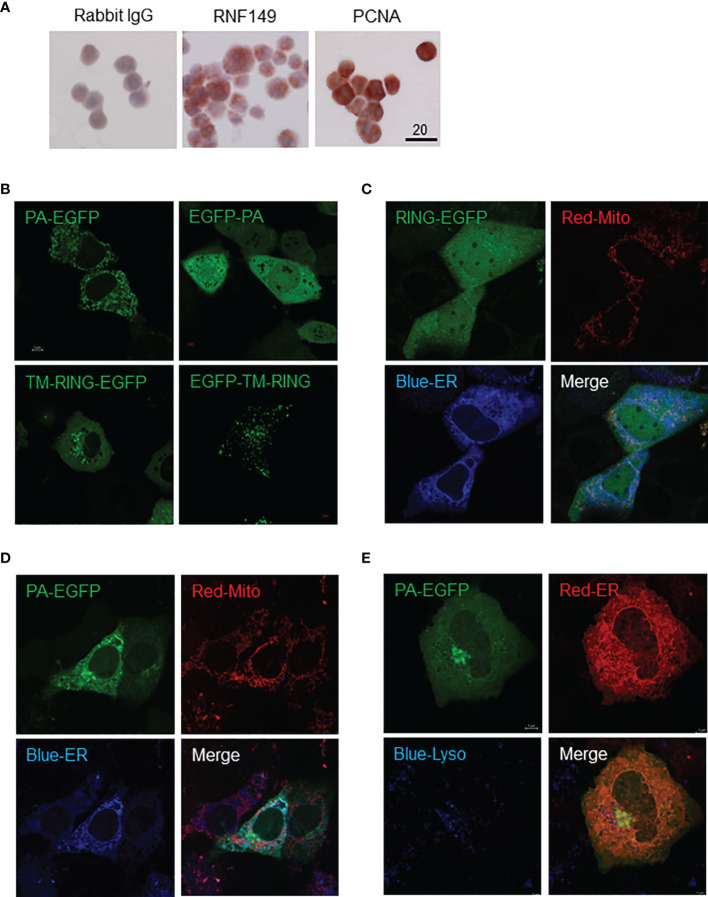
C18-4 cells as a potential model for studying RNF149 function. **(A)** Colorimetric analysis of C18-4 cells positively expressing RNF149 and PCNA. 20μm scale shown. Representative cells shown. **(B)** Single transfection of EGFP-tagged RNF149 isoforms. **(C)** RNF149 is co-localized with ER by PA domain. RING-EGFP vector co-transfected with mitochondria marker (red) and ER marker (blue). **(D)** PA-EGFP vector co-transfected with mitochondria marker (red) and ER marker (blue). Both co-transfections were performed using Lipofectamine™ 3000 (Invitrogen) 24 hours before microscopic observation (according to the manufacturer’s protocol). **(E)** RNF149 is co-localized with ER by PA domain but not the lysosome. PA-type RNF149 isoform is co-transfected with ER marker (red) and lysosome marker [blue; LysoTracker Blue DND-22 (Life Technologies)]. Representative images shown.

C18-4 cells are immortalized spermatogonial cells that exhibit the general properties of type-A spermatogonia and we found these cells to express both RNF149 and PCNA (**Figure 7A**). RNF149 protein expression was found mainly to be cytosolic, in agreement with the cytosolic expression observed in PND8 spermatogonia (data not shown). Given that C18-4 cells express RNF149, this cell line can be used as a potential model to study its role in various germ cell related mechanisms. Four vectors that express either C-termini or N-termini EGFP-tagged VA1 and VA2 forms of RNF149 were transfected into both F9 and C18-4 cell lines independently, with or without the co-transfection of either blue or red ER marking plasmid and lysosome tracker. In C18-4 cells, the location of EGFP on either C-termini or N-termini changed the distribution of RNF149 (**Figure 7B**). Therefore, C-terminal EGFP plasmids were used for subsequent studies. To test where RNF149 isoforms localized in C18-4 cells, two C-terminal EGFP plasmids were co-transfected with markers for the ER and mitochondria. The RING domain-containing RNF149 isoform was widely expressed in C18-4 cell cytoplasm and nucleus (**Figure 7C**), whereas the PA domain-containing isoform co-localized with ER, but not with the mitochondria or lysosome (**Figures 7D, E**). The differential localization of the two variant forms suggests differential functions. Moreover, the PA domain might be required for RNF149 to reside in the ER membrane.

**Figure 8:**
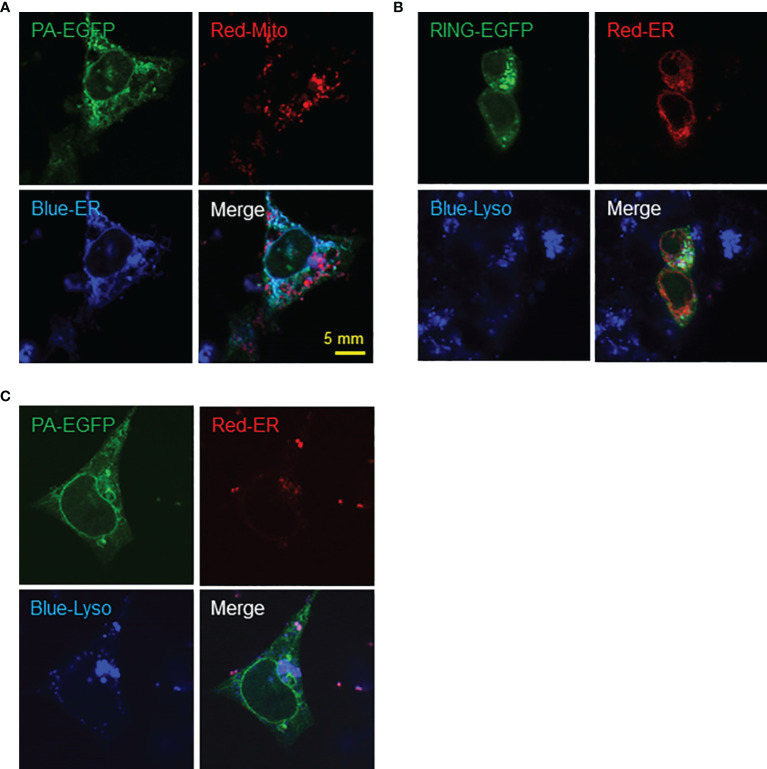
F9 cells as a potential model for studying RNF149 function. **(A)** RNF149 is co-localized with ER by PA domain but not the mitochondria. PA-type RNF149 isoform is co-transfected with ER marker (blue) and mitochondria marker (red). **(B)** PA-domain potentially co-localizes with ER while RING-domain is co-localized with lysosomes. RING-EGFP vector is co-transfected with ER marker (red) and lysosome marker (blue). **(C)** PA-EGFP vector is co-transfected with ER marker (red) and lysosome marker (blue). Representative images shown.

Interestingly, similar expression patterns were observed in F9 cells transfected with PA-EGFP plasmid with markers for ER and mitochondria, where PA-EGFP RNF149 co-localized with the ER rather than mitochondria (**Figure 8A**). When co-transfected with an ER marker and stained with lysosome tracker, PA-EGFP RNF149 did not co-localize with lysosomes in F9 cells, which corresponds to the expression pattern seen in C18-4 cells (**Figure 8B**). However, RING-EGFP RNF149 did not co-localize with ER, but was highly aggregated in lysosomes (**Figure 8C**). These results suggest that in both C18-4 and F9 cells, the PA-domain of RNF149 is a key factor for RNF149 localization in the ER, and that the PA variant protein might have a role in ER, while the RING-domain variant protein might be an essential element for RNF149 localization in the lysosome, related to the protein degradation pathway.

## Discussion

The ubiquitin proteasome system has been widely studied due to its multiple functions in regulating protein degradation, kinase activation, DNA repair, trafficking, translation, and signal pathway activation (12–17). In addition, among the three key enzymes of the UPS, E3 ligases play the most important role as they provide specificity to the entire process (14). RING-type E3 ligases have been reported to be important regulators in many diseases, such as Mdm2, that can ubiquitinate P53 (26), and Skp2, that can degrade c-Myc (27), thus their involvement in various disease states. Some studies have been conducted on transmembrane RING-type E3s such as RNF128 (also called GRAIL) and RNF5, which were reported to participate in cell proliferation and differentiation processes (28). While a functional UPS is required for the regulation of cell proliferation and differentiation in physiological processes such as the formation of ocular lenses, its dysregulation leading to improper proliferation or differentiation is associated with diseases such as cancer and osteoporosis (15, 29, 30). Interestingly, RNF128 was proposed to be linked to cancer and sepsis *via* its role in immunologic tolerance (31).

In contrast, little is known about RNF149, our protein of interest. A study carried in human colon cancer and embryonic kidney cell lines reported that RNF149 indirectly regulated cell differentiation by reducing BRAF, a kinase known for its pro-proliferation function (32). On the other end, a recent study searching germline modifier genes possibly associated with aggressive prostate cancer, by GWAS and human prostate tumor expression data set analyses, identified RNF149 among four genes that could contribute to disease aggressiveness in PC patients. Ectopic overexpression of these genes in *in vitro* cell growth and *in vivo* tumor growth assays found that the overexpression of only one gene, CCDC115, decreased tumor growth, while the other genes had not significant effects in these assays (33). This finding agrees with their observation that RNF149 expression was associated with an increase in disease burden and tumor stage in the patients, whereas CCDC115 high expression was associated with decreased tumor burden (33).

Our previous study showing that RNF149 is strongly expressed in gonocytes and is downregulated during their differentiation to spermatogonia (11), suggests that RNF149 may need to be removed before gonocytes can undergo differentiation to form spermatogonia. This trend is also observed in testes sections, where RNF149 has the highest protein expression in PND2 and PND3 gonocytes, especially in the nucleus, in contrast to its expression in Sertoli cell cytosol in older, pubertal testis. While the full-length rat RNF149 does not have an obvious nuclear signal sequence according to the Nuclear Localization Signal Data Base website (https://rostlab.org/services/nlsdb/), it is possible that RNF149 translocates to the nucleus as part of a protein complex. In addition, RNF149 is found highly expressed in certain cell types in kidney and other organs from PND2 to 35 (puberty), the strongest being in cells from kidney tubules. It is interesting to note that RNF149 profiles reported in the Human Protein Atlas public website (data not shown) were similar to our findings at younger ages in the rat. Indeed, RNF149 was strongly expressed at the surface of spermatocytes and in Sertoli cell cytoplasm in adult human testes, in kidney tubule cells, and in bile duct cells. We also found strong RNF149 expression in adult PND120 Sertoli cell cytoplasm as well as in the cytoplasm of elongated spermatids (data not shown). Thus, our results indicate that RNF149 might be an essential regulator for the postnatal development of different organs. Moreover, its expression varies in testicular germ cells from a nuclear expression in neonatal gonocytes to a cytoplasmic localization in spermatogonia and elongated spermatids, suggesting specific roles in restricted phases of germ cell development. Also of interest is its presence in the cytoplasm of pubertal to adult Sertoli cells, suggesting a potential role in differentiated, but not immature, Sertoli cells.

The analysis of neonatal gonocyte proliferation in response to a mixture of PDGF-BB and 17β-estradiol (P+E), previously found to induce gonocyte proliferation (3, 4), in cells expressing normal levels of RNF149 showed that the expression levels of RNF149 were increased in mock cells simultaneously to PCNA by the proliferative agents, whereas blocking RNF149 expression with siRNA reduced its P+E driven induction by nearly 70% and decreased PCNA induction by more than 50% of the levels found in P+E-treated mock cells. Together with the fact that phospho-ERK, a downstream effector of gonocyte proliferation, was similarly affected by knocking down RNF149, these results suggest that RNF149 may be a positive regulator of gonocyte proliferation. Moreover, the role of RNF149 in neonatal gonocytes appeared specific to this stage of germ cell development, since RNF149 silencing did not affect spermatogonial proliferation. Similarly, the lack of effect of RNF149 silencing on the proliferation of F9 cells suggested that it may not be involved in stem cell proliferation. However, one cannot exclude the possibility that the data obtained with the C18-4 immortalized spermatogonial and the F9 teratoma cell lines might not reflect the function of RNF149 in primary spermatogonia or embryonic stem cells, since these cell lines have deficient cell cycle regulation that could mask a potential role of RNF149 in their proliferation.

A study published in a Chinese journal proposed that RNF149 might be directly involved in cell proliferation *via* degrading CD9 (34). Other studies have linked CD9 to the maintenance of stemness in spermatogonia and its presence in human male germ cells related to their ability to repopulate rodent testes after transplantation (35). It is possible that the target(s) of RNF149 in gonocytes is different from CD9 or that it requires the recruitment of other proteins to affect CD9. Another ubiquitin ligase, RNF144A, was reported to exert a positive effect on cell proliferation in EGF-dependent human cancer and immortalized embryonic cell line models, by maintaining EGFR expression (36). In this study, RNF144A was shown to prolong EGF effects by promoting EGFR ubiquitination, and that RNF144A depletion using CRISPR/Cas9 system decreased EGF-dependent cell proliferation. Subcellular localization studies led to the hypothesis that RNF144A may regulate EGFR transport to intracellular vesicles in EGF-treated cells (36). These findings extend the possibility of RNF proteins regulating cell proliferation not only by regulating the ubiquitination of their target proteins, but also their subcellular localization.

Although very little is known about RNF149, it has been reported to be a transmembrane protein mostly expressed on ER membranes and lysosomes. Besides its potential role in cell proliferation, RNF149 might also participate in the regulation of recycling endosome trafficking. Goliath and Godzilla, two Drosophila members of the PA-TM-RING RNF protein family and their human homologue RNF167, were reported to regulate recycling endosome trafficking *via* ubiquitylation of the VAMP3 (vesicle-associated membrane protein 3) SNARE (soluble N-ethylmaleimide-sensitive factor attachment protein receptor) protein and induce enlargement of EEA1 (early endosome antigen 1)/Rab5-positive early endosomes both *in vitro* and *in vivo* (37). Moreover, a study conducted on LGR5+ stem cells demonstrated that two other PA-TM-RING family proteins RNF43 and ZNRF3, were able to reduce Wnt signals by enhancing endocytosis of Frizzled receptors *via* its ubiquitylation, hence cell growth arrest (38). As a matter of fact, massive activation of Wnt signaling is found in either mice lacking these genes or cancer cells harboring loss-of-function mutations of RNF43 (39). These results implicate a shared regulatory function for PA-TM-RING ubiquitin ligases in intracellular trafficking/sorting and suggest that abrogation of their function may lead to cellular signaling disorder, which can eventually cause cancer.

Our next goal was to generate overexpression vectors coding for rat RNF149 mRNA and to express the recombinant protein in cell lines to determine its subcellular localization. Using publicly available rat RNF149 mRNA sequence, we cloned the gene from rat testes and other tissues. Interestingly, two variant mRNA forms of RNF149, VA1 and VA2, were found in rat testes, liver, and kidney cDNA libraries. Sequence analysis and the positions of start and stop codons showed that VA1 included the sequence of the PA domain but lacked the RING domain. On the other hand, the start codon and stop codon of VA2 defined a sequence including TM and RING domains. Thus, VA2 was referred to as the RING form. These two constructs were then ligated to N1/C2 EGFP vector for mammalian expression of RNF149, to examine their subcellular localization in F9 and C18-4 cells used as models.

Weak RNF149 protein expression was observed in primary rat spermatogonia cytoplasm in PND6-8, in agreement with its expression in mouse C18-4 cells, an immortalized cell line considered as type-A spermatogonia, including spermatogonial stem cells (24). Interestingly, RNF149 expression appeared to be stronger in some but not all C18-4 cells than *in vivo* PND8 spermatogonia, suggesting two subpopulations in growing C18-4 cells, in support of this cell line containing type A spermatogonia at different phase of differentiation, as observed with isolated spermatogonia from juvenile mice (40–42). Alternatively, these different patterns in RRNF149 expression levels could be related to the cells being at different phases of the cell cycle. As a type I transmembrane protein, RNF149 shares common features, such as N-terminal signal peptides (NS) and transmembrane domains, with other members, suggesting that a C-terminal fusion protein of EGFP and RNF149 should not disrupt the N-terminal signal peptides, allowing the fusion protein to remain in the cytoplasm, whereas the fusion of EGFP at the N-terminal might affect its PA-domain function. In RNF protein-related studies, due to the existence of predicted N-terminal signal peptides, EGFP is mostly conjugated to the C-termini. Here, EGFP ligated at the N-termini changed the localization of VA1 and VA2 RNF149, making them either widely spread in the nucleus and cytoplasm, likely due to EGFP hindering the PA domain, resulting in the loss of ability to reside in the ER and other potential sites in the cell, or leading to condensation into smaller spots as seen with VA2. This further suggests that the RING domain participates in intracellular trafficking/sorting. Therefore, to reduce the interference effect caused by EGFP, C-terminal EGFP tagged VA1 and VA2 RNF plasmids will be used in further studies.

In C18-4 and F9 cell lines co-transfected with either the ER, mitochondria, or lysosome marker, VA1 RNF149 was localized in the ER, suggesting a potential function of the PA domain, in agreement with other studies. In contrast, VA2 is localized mainly to lysosomes in F9 cells, which was not observed in C18-4 cells. These results suggest that in both C18-4 and F9 cells, the PA-domain of RNF149 potentially exhibits its function in the ER, while in F9 but not C18-4 cells, the RING-domain may be an essential element for RNF149 translocation to the lysosome, in relation to the protein degradation pathway.

The apparent difference in RING domain localization between F9 cells and C18-4 cells is interesting, since F9 cells correspond to pluripotent embryonic stem cells with both somatic and germ line potentials, whereas C18-4 cells represent more advanced undifferentiated spermatogonia. To date, only BRAF and CD9 are known targets for RNF149, and how they take part and react with both the PA and RING domains of RNF149 remains unclear. Therefore, further studies focused on finding other potential substrates of RNF149 and its actual mechanism of action in these cell lines and gonocytes are required.

In summary, this study demonstrated the potential function of RNF149 in gonocyte development, highlighting the correlation between RNF149 expression and proliferation marker PCNA during PDGF-BB+17β-estradiol co-treatment, the variant forms of RNF149 found in rat tissues, and the potential roles of PA and RING domain-containing variant proteins. Although these studies were not able to fully identify the role of RNF149 and the identity of its substrates in response to proliferation or differential stimulation in gonocytes, C18-4 cells, and F9 cells, they revealed the possibility of RNF149s involvement in gonocyte proliferation and tested the potential use of F9 and C18-4 cell lines as models to study the function of RNF149. The importance of the UPS system in preventing the accumulation of misfolded proteins was recently highlighted, in parallel to the role of autophagy in maintaining cell integrity and functionality (43). Our previous finding that inhibiting proteasome activity impaired gonocyte differentiation (11) and the present study emphasizing RNF149 role in gonocyte proliferation, suggest that multiple UPS enzymes exert different effects in the regulation of these cells and that RNF149 is only a piece of the puzzle. Taken together, the findings that RNF149 expression is induced by proliferating agents, that its silencing decreases proliferation and increases differentiation genes, and that it is downregulated during differentiation, supports the hypothesis that RNF149 plays a role in gonocyte proliferation, while its downregulation may be part of the differentiation process. Moreover, the perturbation of RNF149 function might lead to disorder in membrane protein trafficking and degradation.

## Data Availability Statement

The original contributions presented in the study are included in the article/**Supplementary Material**. Further inquiries can be directed to the corresponding author.

## Ethics Statement

The animal study was reviewed and approved by McGill University Health Centre Animal Care Committee and the Canadian Council on Animal Care.

## Author Contributions

GM contribution includes participate to the study design, performing experiments, data analysis, writing the manuscript, and preparing the table and figures. C-CK was involved in the study design and performing experiments. MC contributed in developing the concept and design of the project, analysing data, preparing and editing the manuscript and figures. All authors contributed to the article and approved the submitted version.

## Funding

This work was supported by a grant from the Natural Sciences and Engineering Research Council of Canada (NSERC) (Discovery Grant # 386038-2013), Canada and by funds from the USC School of Pharmacy to MC. GM was supported by funds from the Division of Endocrinology, Department of Medicine, McGill University, Canada.

## Conflict of Interest

The authors declare that the research was conducted in the absence of any commercial or financial relationships that could be construed as a potential conflict of interest.

## Publisher’s Note

All claims expressed in this article are solely those of the authors and do not necessarily represent those of their affiliated organizations, or those of the publisher, the editors and the reviewers. Any product that may be evaluated in this article, or claim that may be made by its manufacturer, is not guaranteed or endorsed by the publisher.

## Supplementary Material

Supplementary Figure 1 Gene map of RNF149-EGFP plasmid. **(A)** RNF149 VA1-EGFP gene map. **(B)** RNF149 VA2-EGFP gene map.

Supplementary Figure 2 Gene map of EGFP-RNF149 plasmid. **(A)** RNF149 EGFP-VA1 gene map. **(B)** RNF149 EGFP-VA2 gene map.
