# Supplementary figures and images for: Role of the Ubiquitin Ligase RNF149 in the Development of Rat Neonatal Gonocytes
Source: Front Endocrinol (Lausanne). 2022 May 13;13:896507. doi: 10.3389/fendo.2022.896507 (PMC9136010; doi:10.3389/fendo.2022.896507)

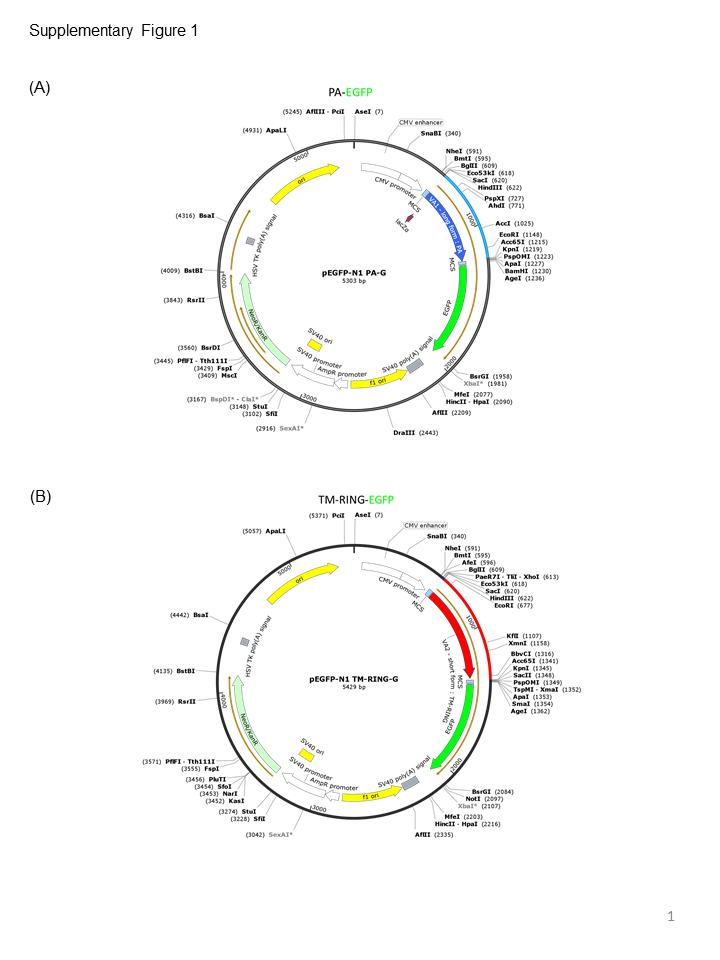

Supplement: Supplementary Figure 1 — Gene map of RNF149-EGFP plasmid. (A) RNF149 VA1-EGFP gene map. (B) RNF149 VA2-EGFP gene map. [file Image_1.tif]

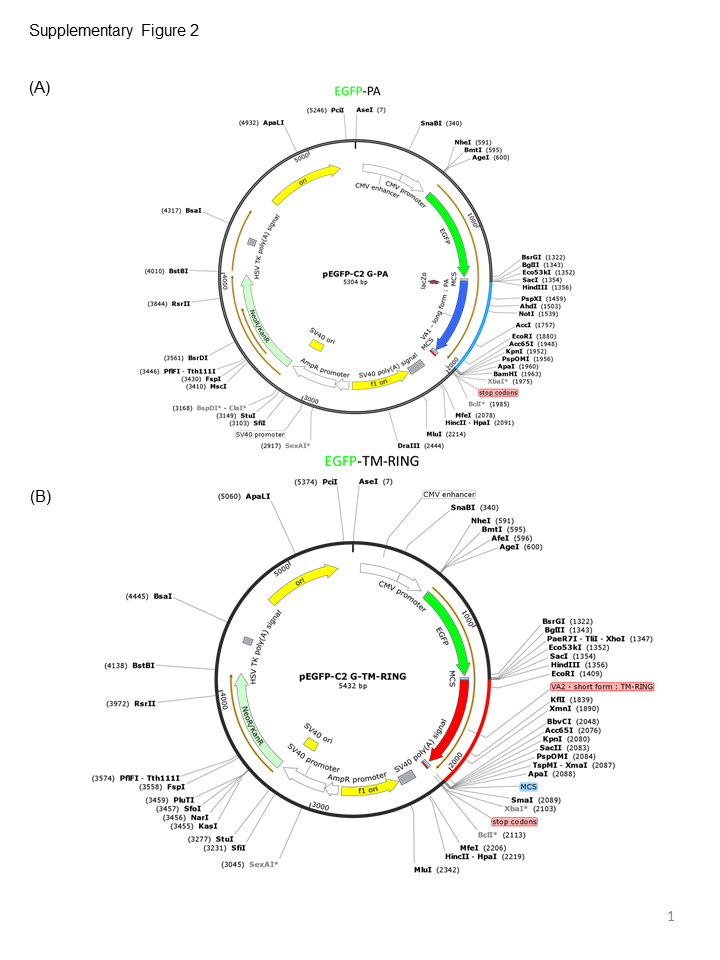

Supplement: Supplementary Figure 2 — Gene map of EGFP-RNF149 plasmid. (A) RNF149 EGFP-VA1 gene map. (B) RNF149 EGFP-VA2 gene map. [file Image_2.tif]
